# Examining Demographic, Geographic, and Temporal Patterns of Melanoma Incidence in Texas From 2000 to 2018: Retrospective Study

**DOI:** 10.2196/67902

**Published:** 2025-05-02

**Authors:** Kehe Zhang, Madison M Taylor, Jocelyn Hunyadi, Hung Q Doan, Adewole S Adamson, Paige Miller, Kelly C Nelson, Cici Bauer

**Affiliations:** 1 Department of Biostatistics and Data Science, School of Public Health, The University of Texas Health Science Center at Houston, E819, 1200 Pressler St., Houston, TX, 77030, United States, 1 7135009581; 2 Center for Spatial-Temporal Modeling for Applications in Population Sciences, School of Public Health, The University of Texas Health Science Center at Houston, Houston, TX, United States; 3 John P. and Kathrine G. McGovern Medical School, The University of Texas Health Science Center at Houston, Houston, TX, United States; 4 Department of Dermatology, Division of Internal Medicine, The University of Texas MD Anderson Cancer Center, Houston, TX, United States; 5 Division of Dermatology, Dell Medical School, The University of Texas at Austin, Austin, TX, United States; 6 Cancer Epidemiology and Surveillance Branch, Texas Department of State Health Services, Austin, TX, United States

**Keywords:** melanoma incidence, melanoma screening, geographic disparity, geospatial analysis, joinpoint regression, demographic variation, temporal trend analysis, stage at diagnosis

## Abstract

**Background:**

Melanoma currently ranks as the fifth leading cancer diagnosis and is projected to become the second most common cancer in the United States by 2040. Melanoma detected at earlier stages may be treated with less-risky and less-costly therapeutic options.

**Objective:**

This study aims to analyze temporal and spatial trends in melanoma incidence by stage at diagnosis (overall, early, and late) in Texas from 2000 to 2018, focusing on demographic and geographic variations to identify high-risk populations and regions for targeted prevention efforts.

**Methods:**

We used melanoma incidence data from all 254 Texas counties from the Texas Cancer Registry (TCR) from 2000 to 2018, aggregated by county and year. Among these, 250 counties reported melanoma cases during the period. Counties with no cases reported in a certain year were treated as having no cases. Melanoma cases were classified by SEER Summary Stage and stratified by the following four key covariates: age, sex, race and ethnicity, and stage at diagnosis. Incidence rates (IRs) were calculated per 100,000 population, and temporal trends were analyzed using joinpoint regression to determine average annual percentage changes (AAPCs) with 95% CIs for the whole time period (2000‐2018), the most recent 10-year period (2009‐2018), and the most recent 5-year period (2014‐2018). Heat map visualizations were developed to assess temporal trends by patient age, year of diagnosis, stage at diagnosis, sex, and race and ethnicity. Spatial cluster analysis was conducted using Getis-Ord Gi* statistics to identify county-level geographic clusters of high and low melanoma incidence by stage at diagnosis.

**Results:**

A total of 82,462 melanoma cases were recorded, of which 74.7% (n=61,588) were early stage, 11.3% (n=9,352) were late stage, and 14% (n=11,522) were of unknown stage. Most cases were identified as males and non-Hispanic White individuals. Melanoma IRs increased from 2000 to 2018, particularly among older adults (60+ years; AAPC range 1.20%-1.84%; all *P* values were <.001), males (AAPC 1.59%; *P*<.001), and non-Hispanic White individuals (AAPC of 3.24% for early stage and 2.38% for late stage; *P*<.001 for early stage and *P* = .03 for late state). Early-stage diagnoses increased while the rates of late-stage diagnoses remained stable for the overall population. The spatial analysis showed that urban areas had higher early-stage incidence rates (*P*=.06), whereas rural areas showed higher late-stage incidence rates (*P*=.05), indicating possible geographic-based differences in access to dermatologic care.

**Conclusions:**

Melanoma incidence in Texas increased over the study time period, with the most-at-risk populations being non-Hispanic White individuals, males, and individuals aged 50 years and older. The stable rates of late-stage melanoma among racial and ethnic minority populations and rural populations highlight potential differences in access to diagnostic care. Future prevention efforts may benefit from increasing access to dermatologic care in areas with higher rates of late-stage melanoma at diagnosis.

## Introduction

Melanoma currently ranks as the fifth leading cancer diagnosis overall and is projected to become the second most diagnosed cancer in the United States by 2040 [1]. Nationally, melanoma incidence rates (IRs) have shown distinct temporal trends across age, sex, race, and ethnicity. Since 2006, IRs have decreased for adolescents and young adults but increased for older adults, with an annual percent change (APC) of 2.5% for individuals older than 65 years between 2006 and 2015 [2,3]. From 2006 to 2021, IRs have increased for non-Hispanic White and Hispanic individuals (APC of 1.7% and 0.6%, respectively) but decreased for non-Hispanic Black individuals (APC of –1.2%) [3]. Non-Hispanic White males older than 50 years have maintained the highest incidence over the last 2 decades [3].

Previous studies have reported that the areal-level social determinants of health (SDoH) are associated with disparities in melanoma incidence, stage at diagnosis, and survival outcomes. For example, using spatial cluster analysis and a multinomial logistic regression model, a study in Florida found that patients with melanoma who live in census tracts with higher percentage of poverty are more likely to have a late-stage diagnosis [4]. Another study examining the national cancer database from 2011 to 2020 used a chi-square test and found that patients from urban areas are more likely to have an early-stage melanoma (*P*<.001) [5]. Similarly, a study in Texas using a multinomial logistic regression model found that patients from counties with persistent poverty (≥20% of residents at or below the federal poverty level for the past two decennial censuses) have higher incidence-based melanoma mortality [6]. Melanoma survival rates vary dramatically depending on stage at diagnosis, with nearly guaranteed 5-year survival for early-stage (localized) diagnoses [3]. However, racial and ethnic minority groups are more likely to be diagnosed with melanoma at advanced stages when compared with stage-matched White patients (chi-square tests with *P*<.001) [3,7,8]. Therefore, understanding how stage-specific melanoma incidence varies across time by patient demographics and geographic location can inform data-driven early detection efforts to improve melanoma morbidity and mortality.

Although Texas had lower all-stage melanoma IRs compared with the national average during 2017 and 2021 (14.9 vs 22.7 cases per 100,000 population), it reported the highest percentage of late-stage cutaneous melanoma diagnoses in the contiguous United States (18.2% in Texas vs 14.1% nationally) [9]. One strategy to shift melanoma detection from late to earlier stages is to increase screening via dermatologists. However, many regions of Texas lack access to dermatologists [10,11]. Alternatively, primary care providers (PCPs) may provide essential skin-cancer detection services [12], yet significant training barriers often preclude early skin cancer diagnosis by PCPs [13]. Geographically targeted education and telementoring efforts to support PCP melanoma diagnosis [14] could potentially enhance early melanoma detection, particularly in areas with a high late-stage melanoma IR. However, the spatial and temporal distribution of stage-based melanoma incidence in Texas has yet to be thoroughly explored to identify these critical locations.

In this study, we analyzed Texas Cancer Registry (TCR) melanoma cases from 2000 to 2018. Using novel data visualizations, we identified trends in melanoma incidence by year of diagnosis, patient demographics, and stage at diagnosis. We also investigated county-level geographic patterns of melanoma incidence across Texas over time. Understanding these trends can guide the development of risk-based interventions to improve melanoma outcomes at the population level.

## Methods

### Data

Melanoma incidence data from 2000 to 2018 were obtained from the TCR and aggregated by 254 Texas counties and year. Among these, 250 counties reported melanoma cases during the period. Counties with no cases reported in a certain year were treated as having no cases. Melanoma cases were categorized by stage at diagnosis using the Surveillance, Epidemiology, and End Results (SEER) summary stage system, which differs from the more clinically oriented National Comprehensive Cancer Network melanoma-specific staging guidelines [10]. While SEER summary stage data categories have changed over time, we organized TCR melanoma cases into three groups: early stage (SEER stages 0 [in situ], 1 [localized], and 2 [regional by direct extension only]), late stage (SEER stages 3 to 5 [regional] and stage 7 [distant]), and unknown (SEER stage 9: unknown, unstaged, and unspecified).

For demographic stratification, we considered 7 age groups (18‐29, 30‐39, 40‐49, 50‐59, 60‐69, 70‐79, and ≥80 years old), 2 sex groups (female and male), and 4 racial and ethnic groups (non-Hispanic White, non-Hispanic Black, Hispanic, and non-Hispanic Others). Annual county-level population estimates stratified by age, sex, and race and ethnicity were obtained from the National Institutes of Health (NIH) SEER county population data [15].

Patient county of residence at the time of diagnosis was classified as rural or urban using the 2013 US Department of Agriculture Rural-Urban Continuum Codes (RUCCs) [16]. RUCCs 1‐3 were classified as urban and RUCCs 4‐9 were classified as rural. Counties were also classified as either with or without persistent poverty using the US Economic Development Administration’s 2021 persistent poverty data [17].

### Incidence Calculation and Trend Analysis

Annual melanoma incidence-based rates were calculated by sex, age, and racial and ethnic groups as described in the “Data” section. Given the substantial variation in melanoma incidence across different age groups, with higher rates typically observed in older age groups, previous analyses often reported age-adjusted rates to allow for more comparable temporal trends across different populations. However, in this study, we adopted a novel data visualization approach using temporal heat maps, which effectively incorporate known confounders such as age and provide a clearer and more intuitive representation of trends [18]. The heat maps display the year of diagnosis (x-axis), age at diagnosis (y-axis), and calculated stage-specific incidence per 100,000 population as a blue (lower rates) to red (higher rates) color gradient. The heat maps used the Akima interpolation method [19] to generate a smoothed surface from observed data points, providing a visually coherent presentation of temporal trends in IRs.

### Spatial Cluster Analysis

We calculated the annual melanoma incidence-based rate for each county in Texas (n=254) using county-specific population data. Spatial cluster analyses were then performed using Getis-Ord Gi* statistics. The Gi* statistic indicates the degree of spatial clustering: positive values indicate that a county and its neighboring areas have higher-than-average rates, while negative values suggest lower-than-average rates. Statistical significance was assessed via Monte Carlo simulation, comparing the observed Gi* values to a reference distribution generated from simulated spatial data. The results categorize counties’ spatial clustering significance as follows: Very High (Gi* stat>0 and *P*<.01), High (Gi* stat>0 and 0.01 *≤ P*<.05), Somewhat High (Gi* stat>0 and 0.05 *≤ P*<.10), Insignificant (*P*>.10), and Low (Gi* stat<0 and *P*<.10). These categorizations allow for the identification of counties with significantly higher or lower melanoma IRs than would be expected by random chance and represent successive thresholds for interpreting spatial clusters without implying a strict ranking of significance. Chi-square tests were used to examine the relationship between spatial clustering categories and urban-rural or poverty status. All data analyses and visualizations were conducted in R (version 4.2.1; R Core Team) [20].

### Joinpoint Trend Analysis

To assess temporal changes in melanoma IR, we performed a state-level joinpoint trend analysis to identify years when significant shifts in trends occured. We calculated the APC in IR using the weighted least-squares method, stratified by stage at diagnosis and among different demographic groups [21]. The APC represents the annual rate of change in IR over a specified period, assuming a constant percentage change each year. For instance, an APC of 2% would indicate that an IR of 100 per 100,000 would increase to 102 per 100,000 in the following year. We allowed for a maximum number of two joinpoints over the 19-year study period. Using the Joinpoint Trend Analysis Software (Joinpoint Regression Program; version 5.3.0.0) from SEER [22], we identified specific years with significant changes in the temporal trends and determined the final number of joinpoints using permutation tests. In addition, we derived a summary measure, the average annual percentage change (AAPC), over three fixed time periods: 2000‐2018 (entire study period), 2009‐2018 (most recent 10 y), and 2014‐2018 (most recent 5 y), based on the joinpoint regression model for the full period from 2000 to 2018. For example, an AAPC of 2% for the 2010‐2018 period would indicate that the IR increased by an average of 2% annually during these years. The 95% CIs for both APC and AAPC were derived using empirical quantile methods.

### Ethical Considerations

The study was approved by the institutional review board (IRB) at the University of Texas Health Science Center at Houston (IRB: HSC-SPH-23‐0483). Melanoma TCR data were obtained from the Texas Department of State Health Services (DSHS) via a formal data request that included an IRB application. Upon IRB approval from the DSHS, a waiver of informed consent was granted because study constitutes secondary research using existing data, involves no more than minimal risk to the participants, and therefore does not require additional consent.

To protect participants’ privacy and confidentiality, all data were de-identified prior to analysis. The dataset included only non-identifiable variables such as year of diagnosis, county of residence at diagnosis, demographic characteristics (e.g., sex, race/ethnicity), and birth year. No names, contact information, or medical record numbers were included. The research team adhered to all DSHS data use agreements and institutional data security protocols. Access to the data was limited to approved study personnel and stored on encrypted, password-protected servers within secure institutional networks.

## Results

### Overview

From 2000 to 2018, the TCR reported 82,462 melanoma cases (Table 1). Among these, 61,588 (74.7%) were diagnosed at an early stage, 9352 (11.3%) at a late stage, and 11,522 (14%) had an unknown stage at diagnosis. The demographic subgroups with the most cases included individuals aged 60‐69 years (18,959 cases, 23.0%), males (49,058 cases, 59.5%), and non-Hispanic White individuals (74,943 cases, 90.9%). The demographic distribution of melanoma cases by stage largely mirrored these overall trends. The racial and ethnic distribution showed that non-Hispanic White individuals dominated both early- and late-stage cases, although their proportion was slightly lower in late-stage cases.

**Figure 1.**
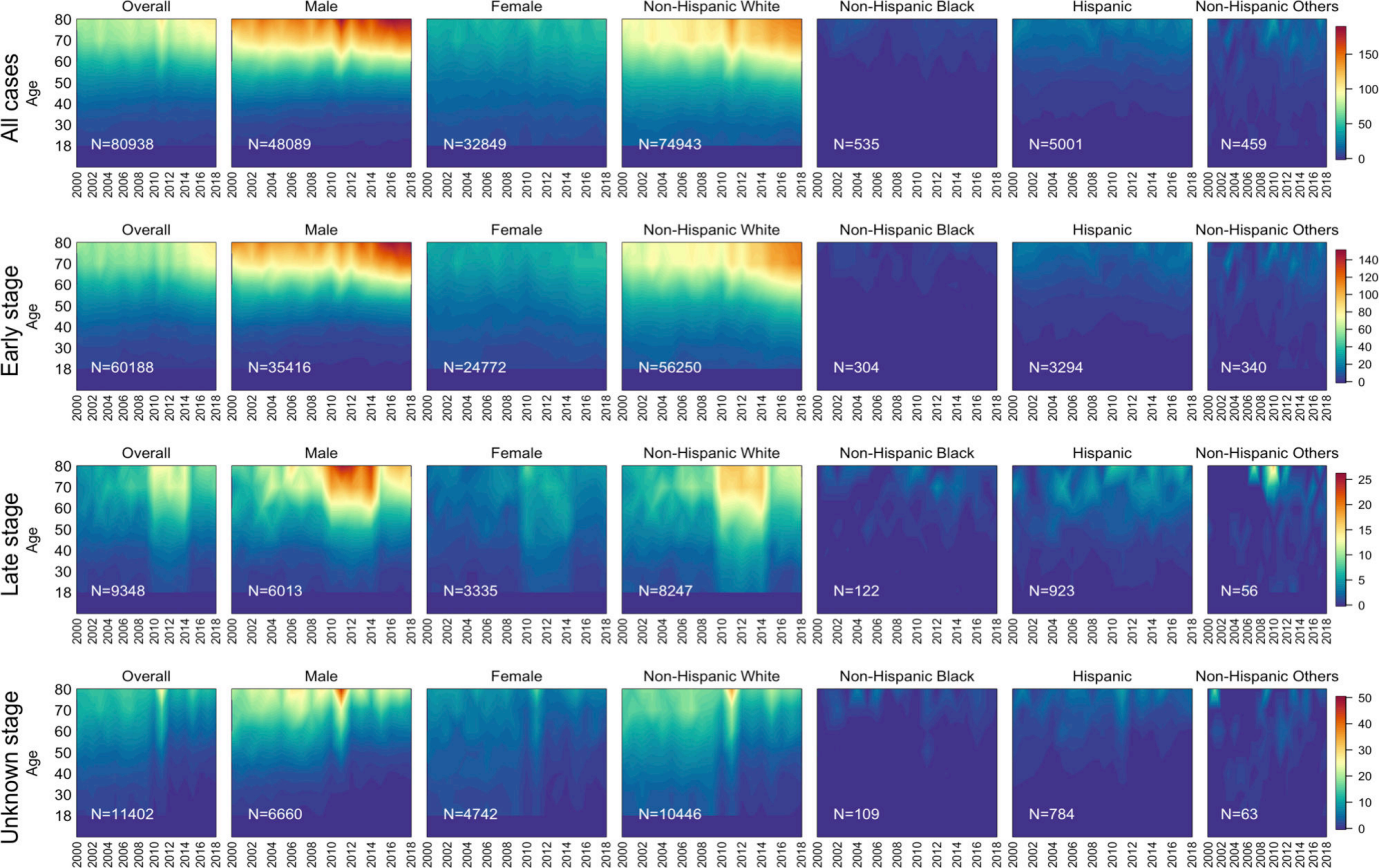
Temporal heat maps presenting melanoma incidence rates (per 100,000 population) by columns of overall population, sex, and racial and ethnicity groups. Each row panel shows a different stage at diagnosis: all cases, early stage, late stage, and unknown stage. Numbers in the lower left corner of each panel indicate the total number of melanoma cases in Texas from 2000 to 2018.

Figure 1 presents temporal heat maps of stage-specific population-adjusted melanoma IRs (cases per 100,000 population) across demographic subgroups (sex, age, and race and ethnicity) from 2000 to 2018 in Texas. When considering all stages, melanoma IRs slightly increased over time to around 90 per 100,000 population. The highest increase was observed in the 80+ age group from 79 in 2000 to 104 in 2018 (AAPC 1.84%, 95% CI 1.27-2.40), while the 70‐79 age group saw the highest increase from 2014‐2018 (AAPC 2.75%, 95% CI 1.84-5.03). Conversely, IRs for the 18‐29 age group declined from 6 in 2014 to 4.6 per 100,000 in 2018 (AAPC 3.05%, 95% CI –3.66 to –2.46). IRs for those aged 30‐49 years remained stable at approximately 10 throughout the study period (Figure 1 and Multimedia Appendix 1).

**Table 1.** Summary of patient demographics by Surveillance, Epidemiology, and End Results (SEER) summary stage system groupings at diagnosis.

Variable	Early stage^a^ (N=61,588), n (%)	Late stage^b^ (N=9,352), n (%)	Unknown stage^c^ (N=11,522), n (%)	All cases (N=82,462), n (%)
Age group (years)				
18‐29	2,196 (3.6)	391 (4.2)	536 (4.7)	3,123 (3.8)
30‐39	4,466 (7.3)	758 (8.1)	927 (8.0)	6,151 (7.5)
40‐49	7,599 (12.3)	1,287 (13.8)	1,589 (13.8)	10,475 (12.7)
50‐59	11,822 (19.2)	2,049 (21.9)	2,149 (18.7)	16,020 (19.4)
60‐69	14,412 (23.4)	2,108 (22.5)	2,439 (21.2)	18,959 (23.0)
70‐79	13,027 (21.2)	1,712 (18.3)	2,156 (18.7)	16,895 (20.5)
≥80	8,066 (13.1)	1,047 (11.2)	1,726 (15.0)	10,839 (13.1)
Sex				
Male	36,300 (58.9)	6,016 (64.3)	6,742 (58.5)	49,058 (59.5)
Female	25,288 (41.1)	3,335 (35.7)	4,780 (41.5)	33,403 (40.5)
Race and ethnicity				
Non-Hispanic White	56,250 (91.3)	8,247 (88.2)	10,446 (90.7)	74,943 (90.9)
Non-Hispanic Black	304 (0.5)	122 (1.3)	109 (0.9)	535 (0.6)
Hispanic	3,294 (5.3)	924 (9.9)	784 (6.8)	5,002 (6.1)
Non-Hispanic Others	340 (0.6)	56 (0.6)	63 (0.5)	459 (0.6)
Unknown	1,400 (2.3)	3 (0.0)	120 (1.0)	1,523 (1.8)

a Early stage: SEER stages 0 (in situ), 1 (localized), and 2 (regional by direct extension only).

b Late stage: SEER stages 3 to 5 (regional) and stage 7 (distant).

c Unknown stage: SEER stage 9: unknown, unstaged, unspecified.

IRs for males appear much higher than females among 50+ years, reaching over 140 per 100,000 in the 70+ age group and over 180 per 100,000 in the 80+ age group by 2018 (Figure 1). In contrast, among younger age groups (18-49), females showed slightly higher rates than males over time. In addition, melanoma incidence increased at a more rapid rate for males from 2009 to 2018, with an AAPC of 3.48% (95% CI 2.49-5.19) compared with 2.47% (95% CI 1.89-3.26) for females (Multimedia Appendix 1).

Stratifying by race and ethnicity, non-Hispanic White individuals displayed the highest rates across all age groups, with noticeable increases over the study period for those aged 50 years and older. The older non-Hispanic White patients (70+ years) showed rates over 130 per 100,000 by 2018. For the 18‐29 age group, non-Hispanic White patients had a slight decrease in IRs from 16.5 per 100,000 in 2000 to 11.4 per 100,000 in 2018. Other racial and ethnic groups, such as non-Hispanic Black and Hispanic individuals, maintained much lower IRs mostly below 30 per 100,000 across all age groups and years (Figure 1).

To facilitate comparison with national statistics, age-adjusted IRs (to the 2000 US standard population) are presented by stage at diagnosis, age, gender, and race and ethnicity in Multimedia Appendix 2.

The incidence of early-stage melanoma has increased more rapidly than late-stage melanoma incidence (Figure 1). Early-stage melanoma temporal trends mirrored those of overall cases, with the highest rates for non-Hispanic White males older than 70 years, reaching 114 cases per 100,000 by 2018. Other racial and ethnic groups displayed minimal variation over time, with rates remaining low throughout the study period (under 10 cases per 100,000 for non-Hispanic Black individuals and under 25 per 100,000 for Hispanic individuals). Late-stage melanoma IRs were lower than early-stage incidence across all demographics. The overall population showed rates below 15 cases per 100,000, with a slight increase over time in older males, peaking around 25 per 100,000 between 2011‐2014 (refer to additional details in Discussion). Non-Hispanic White patients had the highest rates (maximum 16 per 100,000 in the 80+ age group), while non-Hispanic Black and Hispanic patients had consistently low late-stage IRs (mostly below 5 cases per 100,000). The IRs for unknown stage cases remained relatively stable over time across all demographic groups.

When investigating stage by race and ethnicity, early-stage diagnoses predominated across all races and ethnicities. However, Hispanic and non-Hispanic Black individuals had proportionately more late stage at diagnosis melanomas than non-Hispanic White patients (Multimedia Appendix 3). The AAPC for late-stage melanoma cases was similar for Hispanic (AAPC 2.38%, 95% CI 0.53-3.73) and non-Hispanic White patients (AAPC 2.66%, 95% CI –0.98 to 7.17) and higher for non-Hispanic Black patients (AAPC 5.79%, 95% CI 2.61-9.01; Multimedia Appendix 1).

### Spatial Cluster Analysis

**Figure 2.**
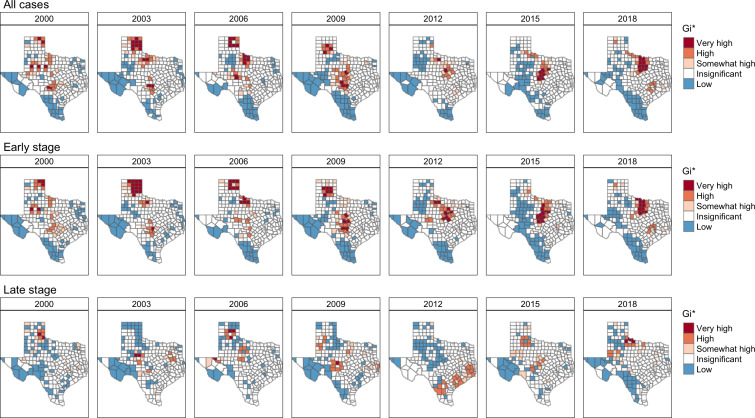
Spatial cluster analysis of melanoma incidence rates (cases per 100,000 population) by melanoma stage at diagnosis (all cases, early stage, and late stage) and selected years from 2000 to 2018 using Gi* statistics. Classifications were defined as follows: very high (Gi*>0 and *P*<.01), high (Gi*>0 and 0.01 *≤ P*<.05), somewhat high (Gi*>0 and 0.05 *≤ P*<.10), insignificant (*P*>.10), and low (Gi*<0 and *P*<.10). Red-shaded areas represent high-incidence clusters, depicting clusters of counties with significantly higher incidence rates compared with the statewide average incidence rate. Blue-shaded areas highlight clusters of counties with significantly lower incidence rates compared with the state average.

Maps showing high- and low-melanoma incidence spatial clusters are presented in Figure 2. When considering all melanoma cases, spatial clusters with significantly higher-than-average melanoma incidence (median IR 42 per 100,000, IQR 23-62) were primarily in northwestern Texas from 2000 to 2006, with a shift to central Texas between 2007 and 2015, and then to counties surrounding Dallas by 2018. Spatial clusters with lower-than-average melanoma incidence were clustered near southern and western Texas (median IR 11, IQR 0-24). The spatial patterns for early-stage and overall melanoma cases were similar with median IRs of 35 per 100,000 (IQR 19-52) in high-incidence spatial clusters, and 6 per 100,000 (IQR 0-17) in low-incidence spatial clusters. However, the spatial clusters for higher-than-average late-stage cases showed distinct patterns with localization to northwestern Texas from 2000 to 2007, a shift toward southeast Texas between 2010 and 2014, and a return to central-northern Texas in 2015.

**Figure 3.**
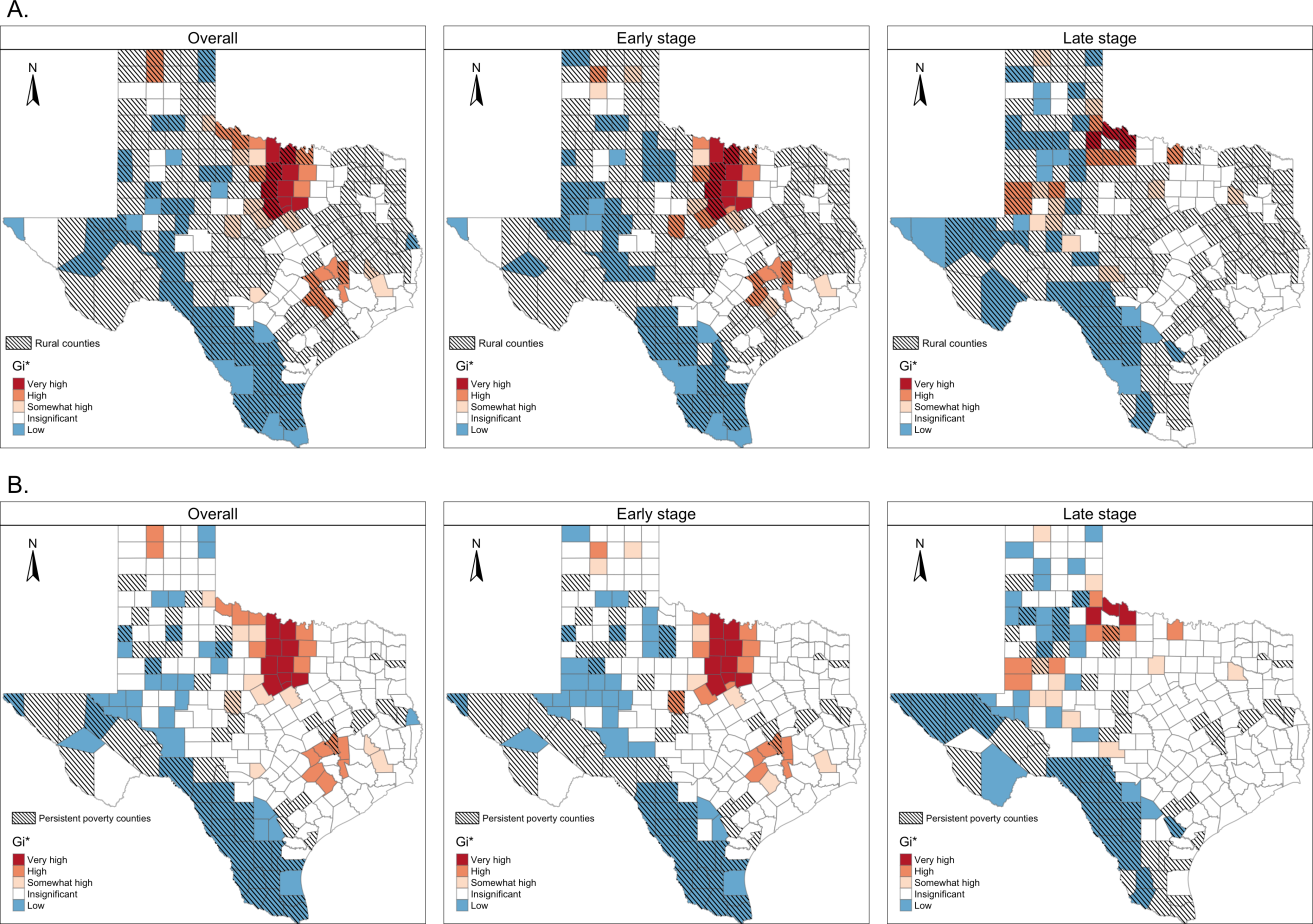
Spatial clusters of melanoma incidence rates overlaid with (A) rural counties and (B) persistent poverty counties in 2018. Classifications were based on Gi* statistics as follows: very high (Gi*>0 and *P*<.01), high (Gi*>0and 0.01 ≤ *P*<.05), somewhat high (Gi*>0 and 0.05 ≤ *P*<.10), insignificant (*P*>.10), and low (Gi*<0 and *P*<.10).

Overlaying the 2018 spatial clusters with rural counties (hatched lines), we observed that clusters of higher-than-average late-stage melanoma incidence significantly overlap with rural areas (*P*=.05; Figure 3A). Similarly, clusters of higher-than-average early-stage melanoma incidence appear to overlap with urban areas (*P*=.06; Figure 3A). In contrast, clusters of lower-than-average incidence for overall, early-stage, and late-stage melanoma appear to overlap with persistent poverty counties (all *P* values were <.001; Figure 3B).

## Discussion

### Principal Findings

Texas has the highest proportion of late-stage melanoma cases relative to the total number of reported melanoma cases in the contiguous United States and is the second most populated state. Identifying the regions and patient populations that disproportionately bear the burden of late-stage melanoma at diagnosis is crucial for developing targeted early detection efforts. Our spatial clustering analysis of Texas revealed that high-incidence clusters of early-stage melanoma were primarily localized in urban, well-resourced areas, whereas high-incidence clusters of late-stage melanoma were concentrated in rural areas . This disparity may be partly explained by the lower density of dermatologists in rural areas [11,23]. Patients with melanoma living in these areas may experience delayed diagnosis [24], need to travel longer distances to receive surgical management, and have decreased melanoma-specific survival [25]. These findings, while observational, highlight the importance of addressing structural disparities in health care access. Therefore, any early detection intervention must be tailored to be feasible in rural, lower-resourced settings.

Melanoma IRs exhibit substantial variation by age, with older patients experiencing significantly higher rates compared with younger patients. Because the age distribution of the population often shifts over time, age-adjusted rates are commonly used to compare trends across different time periods and geographic regions. These adjustments typically use a fixed reference year for the age distribution, which may lack the robustness to fully capture ongoing demographic change. As the population continue to age and shift in age structure, a static reference year may obscure important trends and fail to accurately reflect the current risk landscape. In this study, we used a novel visualization approach using temporal heat maps that directly incorporate age as a variable, providing a more adaptive and precise method for identifying shifts in melanoma incidence trends over time. This visualization highlighted pronounced incidence variations, particularly among late-stage male and non-Hispanic White patients from 2010 to 2014. We identified two TCR data sources which may explain this variation: Texas Health Care Information Collection (THCIC) and eMaRC Plus (Centers for Disease Control and Prevention [CDC]). THCIC, established by the Texas legislature, collects data on health care activities in hospitals and health maintenance organizations [26]. During a pilot from 2010 to 2013, THCIC identified melanoma cases not otherwise reported to TCR, leading to the inclusion of 559 cases in the TCR database, most of which were categorized as unknown stage at diagnosis. The second data source, eMaRC Plus, a software developed by the CDC to receive and process Health Level Seven files from pathology laboratories, was used from 2010 to 2018. eMaRC Plus identified 8,786 cases, with 93.1% being early-stage and 6.8% unknown stage. The peaks in early-stage and unknown-stage incidence observed in 2011 and onward may be attributed to these two data sources. However, the increase in late-stage incidence from 2010 to 2014 could not be fully explained by these data sources, suggesting that temporary reporting inconsistencies may warrant further investigation to fully understand their impact on the identified temporal trends.

Despite these data source differences, descriptive trends also revealed that older non-Hispanic White men comprise the majority of late-stage melanoma cases at diagnosis. This specific demographic provides a clear target cohort for refining early melanoma detection efforts. Given that older men generally have lower rates of skin self-awareness [27], promoting early detection through their family members or PCPs may offer greater opportunity. Our analysis of race and ethnicity revealed that, while most melanoma cases were diagnosed at early stages across all groups, Hispanic and non-Hispanic Black patients experienced proportionately more late stage at diagnosis melanomas than non-Hispanic White counterparts. Although the absolute number of melanoma cases is small compared with other cancers, these findings reinforce the concept that melanoma can impact individuals of all races and ethnicities.

### Strengths and Limitations

This study has several strengths and limitations. A key strength is the innovative visualization approach, which enabled a comprehensive analysis of temporal trends, stratified by age, sex, race and ethnicity, and stage at diagnosis. The use of TCR data allowed for the inclusion of patient residential information at the time of diagnosis, as well as additional data captures from pilot studies that would otherwise be unavailable. Furthermore, the geospatial analysis provided a comprehensive examination of patterns across all cases and by specific stages, offering valuable insights that could guide future interventions and educational efforts. The study also presents several limitations, particularly the large proportion of cases with an unknown stage at diagnosis. More precise data on these cases would enable better stage classification and improve the stage-specific analyses. In addition, the TCR data has limitations in capturing SDoH, which, if included, could provide a deeper understanding of the health disparities associated with melanoma outcomes.

### Conclusions

Our study provides valuable guidance for future early melanoma detection efforts. Such efforts must be feasible in rural, lower-resourced areas of the state and focus on patients at highest risk of late-stage melanoma at diagnosis. Multimodal approaches, which combine foundational dermatology training for interested PCPs [14], telementoring to support PCP incorporation of skin cancer detection examinations into practice [28], and efficient store-and-forward eConsults [29] to reduce dermatology access gaps, offer promising pathways to improve early melanoma detection in low-resource settings.

## Supplementary material

10.2196/67902 Multimedia Appendix 1 Average annual percent change (AAPC) in melanoma incidence rates and corresponding 95% CI, stratified by stage at diagnosis (overall, early, and late) and demographic factors (age, sex, and race and ethnicity), for the time intervals 2000-2018, 2009-2018, and 2014-2018. Results were based on Joinpoint regression analysis for the time interval of 2000-2018.

10.2196/67902 Multimedia Appendix 2 Age-adjusted melanoma incidence rates (cases per 100,000 population) by stage at diagnosis and demographics groups, standardized to the 2000 US population.

10.2196/67902 Multimedia Appendix 3 Melanoma incidence rates by race and ethnicity, and stage at diagnosis from 2000 to 2018.

## Abbreviations

AAPC: average annual percentage change
APC: annual percent change
CDC: Centers for Disease Control and Prevention
DSHS: Department of State Health Services
IR: incidence rate
IRB: institutional review board
NIH: National Institutes of Health
PCP: primary care provider
RUCC: Rural-Urban Continuum Code
SDoH: social determinants of health
SEER: Surveillance, Epidemiology, and End Results
TCR: Texas Cancer Registry
THCIC: Texas Health Care Information Collection
