# Supplementary material for: Examining Demographic, Geographic, and Temporal Patterns of Melanoma Incidence in Texas From 2000 to 2018: Retrospective Study
Source: JMIR Cancer. 2025 May 2;11:e67902. doi: 10.2196/67902 (PMC12064134; doi:10.2196/67902)
Supplement: Multimedia Appendix 1 [file cancer-v11-e67902-s001.docx]

**Multimedia Appendix 1.** Average Annual Percent Change (AAPC) in melanoma incidence rates and associated 95% confidence intervals (CI) by stage (overall, early and late) and demographics (age, sex and race and ethnicity) for time interval 2000-2018, 2009-2018 and 2014-2018. Results were based on the Joinpoint regression analysis for the time interval of 2000-2018.

| **Demographic Variable (Stage)** | **2000-2018 Fixed Interval** | **2009-2018 Fixed Interval** | **2014-2018 Fixed interval** |
| --- | --- | --- | --- |
|  | AAPC (95% CI) | AAPC (95% CI) | AAPC (95% CI) |
| **Age (Overall)** |  |  |  |
| 18-29 | -3.05* (-3.66, -2.46) | -3.05* (-3.66, -2.46) | -3.05* (-3.66, -2.46) |
| 30-39 | -0.80* (-1.41, -0.11) | -0.12 (-0.89, 1.25) | -0.12 (-1.05, 1.25) |
| 40-49 | -1.14* (-1.65, -0.69) | -0.01 (-0.80, 0.74) | 1.83 (-0.50, 4.48) |
| 50-59 | 0.05 (-0.21, 0.29) | 1.29* (0.79, 1.74) | 1.60* (0.88, 2.66) |
| 60-69 | 1.20* (0.59, 1.81) | 2.26* (1.30, 3.21) | 2.52* (1.33, 5.69) |
| 70-79 | 1.65* (1.22, 2.08) | 2.53* (1.82, 3.22) | 2.75* (1.84, 5.03) |
| 80+ | 1.84* (1.27, 2.40) | 2.64* (1.74, 3.54) | 2.64* (1.74, 5.36) |
| **Sex (Overall)** |  |  |  |
| Female | 0.94* (0.70, 1.16) | 2.47* (1.89, 3.26) | 2.47* (1.89, 2.87) |
| Male | 1.59* (1.22, 1.95) | 3.48* (2.49, 5.19) | 3.10* (2.41, 3.73) |
| **Race/Ethnicity (Early)** |  |  |  |
| Non-Hispanic White | 3.24* (2.75, 3.69) | 5.66* (4.78, 6.45) | 7.07* (5.37, 10.04) |
| Non-Hispanic Black | 4.46 (-0.08, 7.55) | 4.46 (-0.08, 7.55) | 1.14 (-11.46, 4.64) |
| Hispanic | 1.44* (0.24, 2.54) | 5.38* (3.38, 7.32) | 7.70* (3.93, 14.57) |
| **Race/Ethnicity (Late)** |  |  |  |
| Non-Hispanic White | 2.38* (0.53, 3.73) | -0.80 (-4.12, 1.49) | -8.48* (-15.01, 4.46) |
| Non-Hispanic Black | 5.79* (2.61, 9.01) | 5.79* (2.61, 9.01) | 5.79* (2.61, 9.01) |
| Hispanic | 2.66 (-0.98, 7.17) | -0.10 (-6.51, 4.64) | -4.36 (-19.3, 4.81) |

Abbreviations: AAPC: average annual percent of change; CI: confidence intervals.

*Significant change in melanoma incidence rates (The AAPC is statistically significantly different from zero; p-value < 0.05).
