# Supplementary figures and images for: Examining Demographic, Geographic, and Temporal Patterns of Melanoma Incidence in Texas From 2000 to 2018: Retrospective Study
Source: JMIR Cancer. 2025 May 2;11:e67902. doi: 10.2196/67902 (PMC12064134; doi:10.2196/67902)

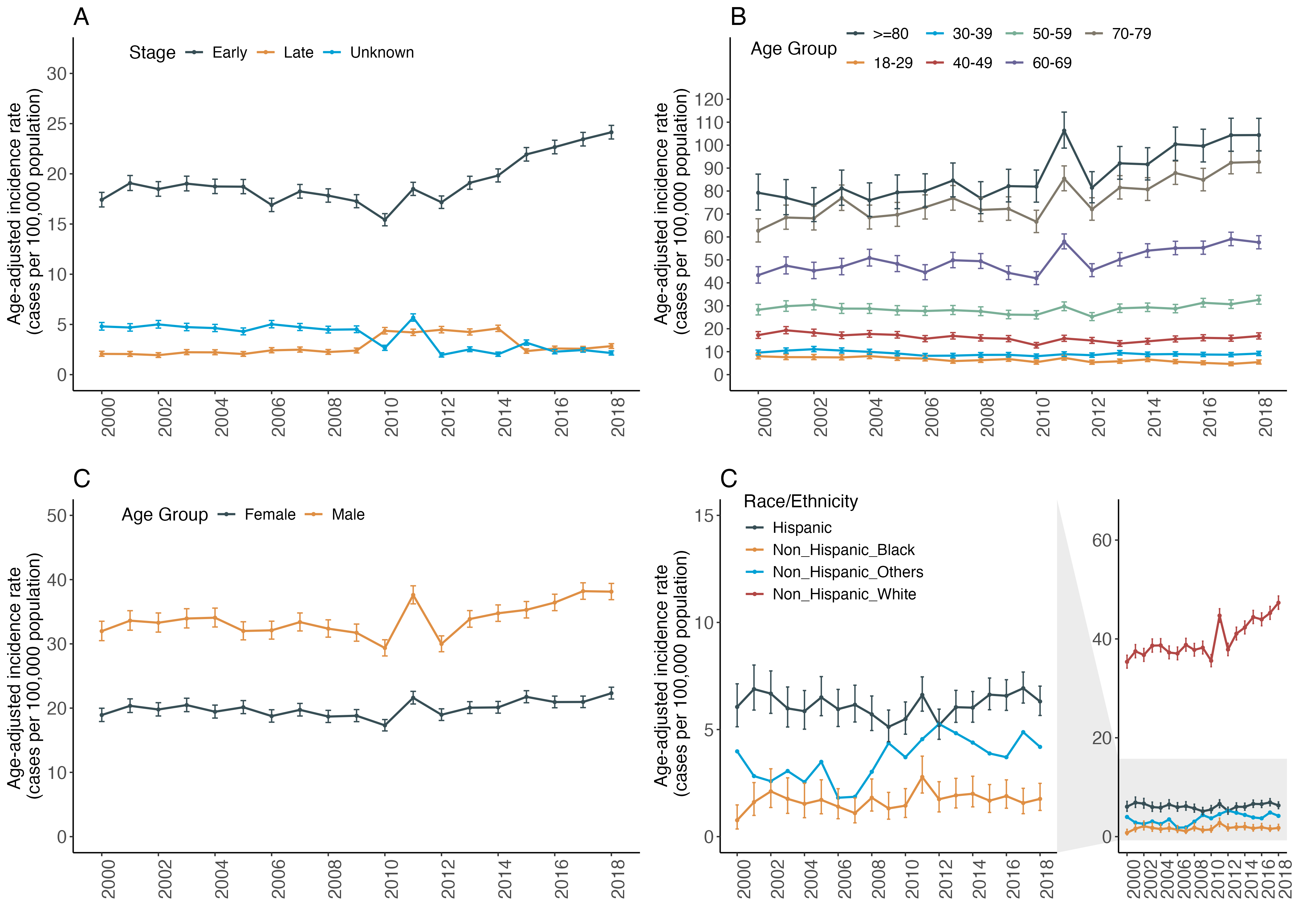

Supplement: Multimedia Appendix 2 [file cancer-v11-e67902-s002.png]

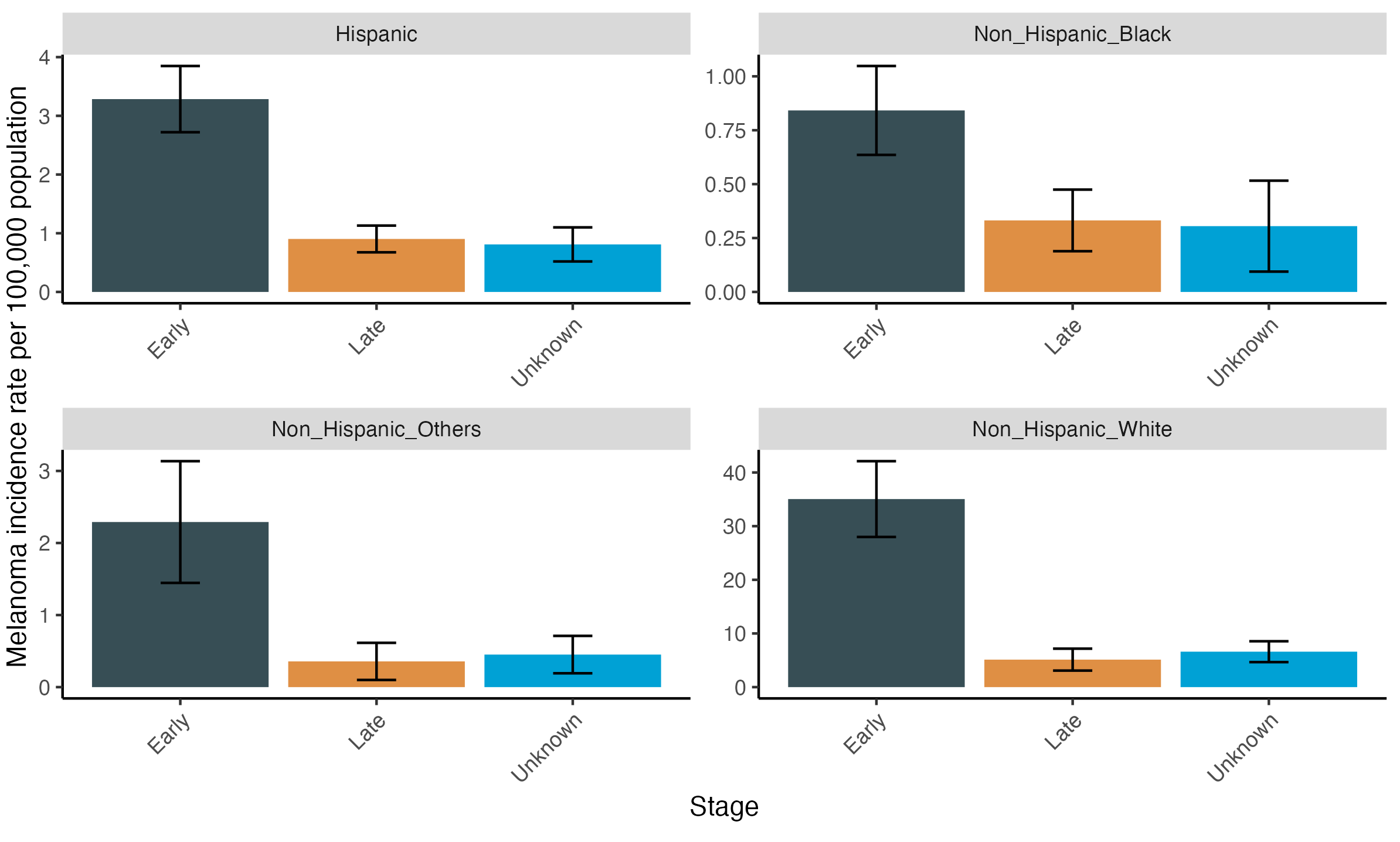

Supplement: Multimedia Appendix 3 [file cancer-v11-e67902-s003.png]
